# New Saffold Cardiovirus in Children, China

**DOI:** 10.3201/eid1506.090109

**Published:** 2009-06

**Authors:** Zi-Qian Xu, Wei-Xia Cheng, Hong-Mei Qi, Shu-Xian Cui, Yu Jin, Zhao-Jun Duan

**Affiliations:** State Key Laboratory for Molecular Virology and Genetic Engineering, Beijing, People’s Republic of China (Z.-Q. Xu, S.-X.Cui, Z.-J.Duan); The First Hospital of Lanzhou University, Lanzhou, People’s Republic of China (W.-.X. Cheng, H.-M. Qi, Y. Jin)

**Keywords:** Viruses, Saffold cardiovirus, children, China, letter

**To the Editor:** A new member of the genus *Cardiovirus*, termed *Saffold virus* (SAFV), was discovered recently in stool specimens and nasopharyngeal aspirate samples from patients with fever of unknown origin, respiratory symptoms, or gastroenteritis; these have been considered the first documented reports of cardiovirus infection in humans (*1*–*4*). However, the epidemiologic characteristics and pathogenic role of the virus are not fully understood.

From July 2006 through June 2008, stool specimens were collected from 631 hospitalized children with diarrhea and 161 asymptomatic controls in Lanzhou, People’s Republic of China. All children were <5 years of age (median age 8 months, range 0–60 months). Diarrhea was defined as >3 loose stools in the previous 24–72 h. Controls were asymptomatic children who had been brought to the First Hospital of Lanzhou University Pediatric Primary Care Center for a routine checkup and had not had fever, diarrhea, vomiting, or a respiratory illness in the previous 3 weeks. The stool specimens were then transported to the Chinese Center for Disease Control and Prevention, Beijing, to undergo screening for common enteric viruses. The specimens were tested for rotavirus by using a commercially available ELISA kit (IDEIA Rotavirus; DAKO, Glostrup, Denmark), and PCR and reverse transcription PCR (*5*) were used to screen for other common enteric viruses, including norovirus, sapovirus, astrovirus, and adenovirus.

Viral RNA and DNA were extracted from 140 µL of 10% fecal suspension in phosphate-buffered saline by using QIAamp Viral RNA Mini Kit (QIAGEN, Hilden, Germany); viral RNA and DNA was supposed to be extracted simultaneously, according to the manufacturer’s instructions. Extracts of nucleic acid were tested for SAFV by a nested PCR that targeted the 5′ untranslated region (UTR) gene as described by Drexler et al. (*4*). The viral protein 1 (VP1) gene from positive samples was amplified as described by Chiu et al. (*3*). Positive bands were cloned and sequenced in both directions.

By confirming sequences of the 5′ UTR gene, 3 (0.5%) specimens from the 631 children with diarrhea (LZ50419, LZ52903, LZ53879) and 1 (0.6%) from the 161 asymptomatic children (LZ53010) were found to be positive for SAFV. Of the 4 positive specimens, 2 were collected in October, 1 in September, and 1 in June. The median age of the 4 patients with positive specimens was 6 months (range 2–25 months). Viral co-infection was detected in the 3 children with diarrhea who had SAFV-positive specimens; 2 were co-infected with rotavirus and 1 with norovirus. No co-infection was detected in the asymptomatic child with SAFV-positive results.

The 0.5% detection rate of SAFV in children with diarrhea in our study is lower than the 1.2% reported by Chiu et al. (*3*). One possible reason could be that the patients in our study were younger (median age 8 months), but in other studies, the median age was 20 months for all patients with confirmed cases. Nonetheless, the seasonal distribution of the positive cases in our study is in accordance with the result of Drexler et al. (*4*), namely, in late summer and early fall.

The 5′ UTR sequences of the 4 positive samples were deposited in GenBank (accession nos. FJ586238, FJ586239, FJ610244, and FJ623968). After several trials, only the VP1 sequence of sample LZ50419 was amplified (accession no. FJ586240). The obtained sequences were analyzed by using the DNASTAR software package (DNASTAR, Madison, WI, USA). A BLAST search (http://blast.ncbi.nlm.nih.gov/Blast.cgi) demonstrated that that 5′ UTR sequences of the 4 positive samples— LZ50419, LZ52903, LZ53010, and LZ53879—had nucleotide identity of 96%, 91%, 96%, and 95% to the SAFV prototype strains (SAFV 1 California/81, EF165067.2), respectively; the VP1 sequence of sample LZ50419 had a nucleotide identity of 87% and amino acid identity of 97% with the SAFV1 California/81. Phylogenetic analysis (Figure) showed that the SAFV found in China clustered with the strain isolated in the United States in 1981. This finding suggests that the 1981 VP1 lineage was still circulating. More study is needed to address genetic variation of this lineage.

**Figure:**
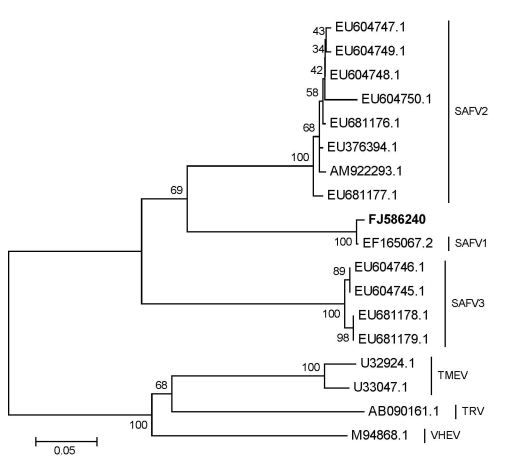
Phylogenetic relationships of deduced partial viral protein 1 amino acid sequences. Phylogenetic analyses using MEGA version 3.1 (www.megasoftware.net) and the neighbor-joining algorithm calculated by the Poisson correction model were based on alignment of 289 amino acids. The new strain from this study is shown in **boldface***.* The scale bar indicates the genetic distance of 0.05 substitution/site. SAFV, Saffold virus; TMEV, Theiler’s murine encephalomyelitis virus; TRV, Thera virus; VHEV, Vilyuisk human encephalomyelitis virus.

Our finding of SAFV in children hospitalized with diarrhea in China suggests that the virus is distributed worldwide. The detection of the virus in the asymptomatic control in our study is also noteworthy. Fisher exact test results showed no significant difference in the detection rate between the case group and the control group. The concurrent detection of SAFV and other enteric viruses raises concern over a causative role of SAFV in human gastroenteritis. The current study does not suggest that SAFV has any association with acute enteritis based on a statistical analysis. Also, as with several other novel viruses discovered recently, SAFV has not been associated with any clinically relevant disease in humans, although it has been isolated from cell cultures (*4*). Thus, we presume that SAFV is only a gastroenteric passenger because most picornaviruses are associated with enteric infection and with a known fecal–oral route of transmission. More comprehensive studies are needed to ascertain whether SAFV has any clinical relevance.
